# (*E*)-1-(4-Bromo­phen­yl)-3-(2,4,6-trimethoxy­phen­yl)prop-2-en-1-one^1^

**DOI:** 10.1107/S1600536809010496

**Published:** 2009-03-28

**Authors:** Suchada Chantrapromma, Thitipone Suwunwong, Chatchanok Karalai, Hoong-Kun Fun

**Affiliations:** a Crystal Materials Research Unit, Department of Chemistry, Faculty of Science, Prince of Songkla University, Hat-Yai, Songkhla 90112, Thailand; b X-ray Crystallography Unit, School of Physics, Universiti Sains Malaysia, 11800 USM, Penang, Malaysia

## Abstract

The mol­ecule of the title chalcone derivative, C_18_H_17_BrO_4_, is twisted, the dihedral angle between the 4-bromo­phenyl and 2,4,6-trimethoxy­phenyl rings being 39.17 (6)°. The three meth­oxy groups are oriented in two different conformations whereby two meth­oxy groups are coplanar, whereas the third is twisted with respect to the attached benzene ring [C—O—C—C torsion angles of −2.84 (18), −2.80 (18) and −9.31 (18)°]. Weak intra­molecular C—H⋯O inter­actions generate two *S*(5) and one *S*(6) ring motifs. In the crystal structure, mol­ecules are linked into supra­molecular sheets parallel to the *bc* plane by weak C—H⋯O inter­actions. These sheets are stacked along the *a* axis. The crystal structure is further stabilized by weak C—H⋯π inter­actions.

## Related literature

For bond-length data, see: Allen *et al.* (1987 ▶). For hydrogen-bond motifs, see: Bernstein *et al.* (1995 ▶). For a related structure, see: Suwunwong *et al.* (2009 ▶). For background to and applications of chalcones, see: Fayed & Awad (2004 ▶); Jung *et al.* (2008 ▶); Patil & Dharmaprakash (2008 ▶); Prasad *et al.* (2008 ▶); Sens & Drexhage (1981 ▶) and Xu *et al.* (2005 ▶). For the stability of the temperature controller used in the data collection, see Cosier & Glazer, (1986 ▶).
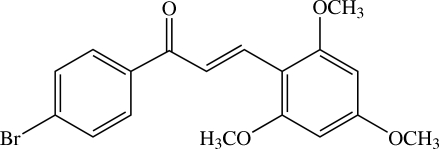


## Experimental

### 

#### Crystal data

C_18_H_17_BrO_4_
*M* *_r_* = 377.22
Triclinic, 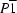

*a* = 6.3690 (1) Å
*b* = 9.2553 (1) Å
*c* = 14.1884 (2) Å
α = 104.397 (1)°
β = 93.748 (1)°
γ = 98.799 (1)°
*V* = 795.88 (2) Å^3^
*Z* = 2
Mo *K*α radiation
μ = 2.60 mm^−1^
*T* = 100 K
0.27 × 0.20 × 0.15 mm

#### Data collection

Bruker APEXII CCD area-detector diffractometer
Absorption correction: multi-scan (*SADABS*; Bruker, 2005 ▶) *T*_min_ = 0.541, *T*_max_ = 0.701
16214 measured reflections
4607 independent reflections
4118 reflections with *I* > 2σ(*I*)
*R*_int_ = 0.023

#### Refinement

*R*[*F* ^2^ > 2σ(*F* ^2^)] = 0.024
*wR*(*F* ^2^) = 0.060
*S* = 1.02
4607 reflections
276 parameters
All H-atom parameters refined
Δρ_max_ = 0.42 e Å^−3^
Δρ_min_ = −0.23 e Å^−3^

### 

Data collection: *APEX2* (Bruker, 2005 ▶); cell refinement: *SAINT* (Bruker, 2005 ▶); data reduction: *SAINT*; program(s) used to solve structure: *SHELXTL* (Sheldrick, 2008 ▶); program(s) used to refine structure: *SHELXTL*; molecular graphics: *SHELXTL*; software used to prepare material for publication: *SHELXTL* and *PLATON* (Spek, 2009 ▶).

## Supplementary Material

Crystal structure: contains datablocks global, I. DOI: 10.1107/S1600536809010496/sj2593sup1.cif

Structure factors: contains datablocks I. DOI: 10.1107/S1600536809010496/sj2593Isup2.hkl

Additional supplementary materials:  crystallographic information; 3D view; checkCIF report

## Figures and Tables

**Table 1:** Hydrogen-bond geometry (Å, °)

*D*—H⋯*A*	*D*—H	H⋯*A*	*D*⋯*A*	*D*—H⋯*A*
C5—H5⋯O3^i^	0.925 (18)	2.497 (18)	3.3572 (17)	154.9 (15)
C8—H8⋯O4	0.920 (18)	2.268 (18)	2.8103 (16)	117.2 (14)
C9—H9⋯O1	0.936 (18)	2.449 (19)	2.8128 (17)	103.1 (13)
C9—H9⋯O2	0.936 (18)	2.269 (19)	2.6960 (16)	107.1 (14)
C18—H18*A*⋯O1^ii^	0.968 (19)	2.566 (19)	3.4518 (18)	152.1 (15)
C17—H17*C*⋯*Cg*1^iii^	0.971 (17)	2.754 (18)	3.6601 (14)	155.6 (14)

Symmetry codes: (i) 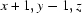; (ii) 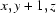; (iii) 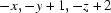. *Cg*1 is the centroid of the C10–C15 ring.

**Fig. 1.**
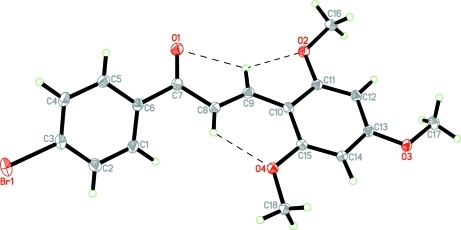
The molecular structure of the title compound, with 50% probability displacement ellipsoids and the atom-numbering scheme. Intramolecular hydrogen bonds are shown as dashed lines.

**Fig. 2.**
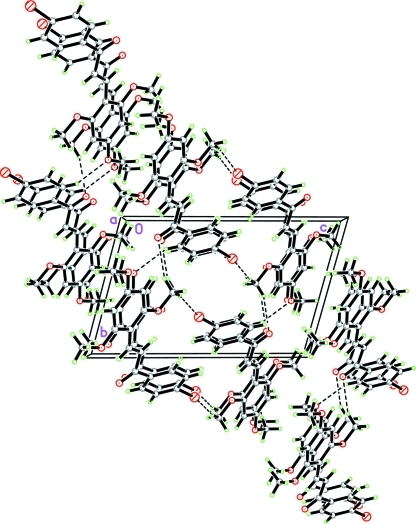
The crystal packing of the title compound, showing supramolecular sheets. Hydrogen bonds are shown as dashed lines.

## supplementary crystallographic information

### Comment

Chalcones are compounds which have a wide range of applications such as in non-linear optical (Patil & Dharmaprakash, 2008) and electro-active fluorescent materials (Jung *et al.*, 2008) or materials with various biological activities (Prasad *et al.*, 2008). Fluorescent compounds are used for various applications such as fluorescent dyes (Fayed & Awad, 2004), light-emitting diodes, LEDs, (Sens & Drexhage, 1981) and fluorescent probes (Xu *et al.*, 2005). In general, fluorescent materials are aromatic compounds which are conjugated with a double bond and/or aliphatic/alicyclic carbonyl groups typified by the structures of chalcone derivatives. These interesting properties prompted us to synthesize the title chalcone derivative in order to study its fluorescent properties and to compare these properties with those of a closely related structure (Suwunwong *et al.*, 2009). We report here the crystal structure of the title compound (I).

The molecule of (I) in Fig. 1 exists in an *E* configuration with respect to the C8//db C9 double bond [1.3486 (18) Å]. The molecule is twisted with the interplanar angle between the 4-bromophenyl and the 2,4,6-trimethoxyphenyl rings being 39.17 (6)° compared to the corresponding angle of 44.18 (6)° between the 4-bromophenyl and the 3,4,5-trimethoxyphenyl ring in the closely related structure, 2*E*-1-(4-bromophenyl)-3-(3,4,5-trimethoxyphenyl) prop-2-en-1-one (II) (Suwunwong *et al.*, 2009). Atoms O1, C6, C7 and C8 lie on the same plane with a maximum deviation of -0.001 (1) Å for atom C7 and the mean plane through them makes dihedral angles of 27.54 (7)° and 12.35 (7)° with the 4-bromophenyl and the 2,4,6-trimethoxyphenyl rings, respectively. The three methoxy groups of the 2,4,6-trimethoxyphenyl unit adopt two different orientations. The C13 and C15 methoxy groups are co-planar with the attached benzene ring with torsion angles C17–O3–C13–C12 = -2.84 (18)° and C18–O4–C15–C14 = -2.80 (18)° whereas the C11 group is twisted with a torsion angle C16–O2–C11–C12 = -9.31 (18)° indicating (-)-*syn*-periplanar conformations. Weak intramolecular C9—H9···O1 and C9—H9···O2  interactions generate S(5) ring motifs whereas a weak intramolecular C8—H8···O4 interaction generates an S(6) ring motif (Bernstein *et al.*, 1995) (Table 1). The different substitutional positions of the three methoxy groups in 2,4,6-trimethoxyphenyl of (I) compared to the 3,4,5-trimethoxy groups in (II) (Suwunwong *et al.*, 2009), produced different weak intramolecular C—H···O interactions especially the weak C9—H9···O2 and C9—H9···O4 intramolecular interactions which help the molecule of (I) to be less twisted. Bond distances in the molecule are normal (Allen *et al.*, 1987) and are comparable with those in the closely related structure (Suwunwong *et al.*, 2009).

In the crystal packing (Fig. 2), molecules are linked by weak intermolcular C5—H5···O3 (symmetry code: 1 + *x*, -1 + *y*, *z*) and C18—H18A···O1 (symmetry code: *x*, 1 + *y*, *z*) interactions (Table 1) into supramolecular sheets parallel to the *bc* plane. These sheets are stacked along the *a* axis. The crystal structure is further stabilized by weak C—H···π interactions (Table 1); *Cg*1 is the centroid of the C10–C15 ring.

### Experimental

The title compound was synthesized by the condensation of 2,4,6-trimethoxybenzaldehyde (0.4 g, 2 mmol) with 4-bromoacetophenone (0.4 g, 2 mmol) in ethanol (30 ml) in the presence of 10% NaOH(aq) (5 ml). After stirring for 4 h in an ice bath (278 K), a pale yellow solid appeared after leaving the mixture at room temperature for 4 h. The resulting pale yellow solid was collected by filtration, washed with distilled water, dried and purified by repeated recrystallization from acetone. Pale yellow block-shaped single crystals of the title compound suitable for *x*-ray structure determination were recrystalized from acetone/ethanol (1:1 *v*/*v*) by slow evaporation of the solvent at room temperature over several days, Mp. 425–426 K.

### Refinement

All H atoms were located in a difference maps and refined isotropically. *U*_iso_ = 1.2*U*_eq_(C) for aromatic and CH and *U*_iso_ = 1.5*U*_eq_(C) for CH_3_ atoms. The highest residual electron density peak is located at 0.70 Å from C15 and the deepest hole is located at 0.63 Å from C15.

### Crystal data

**Table:**

C_18_H_17_BrO_4_	*Z* = 2
*M**_r_* = 377.22	*F*(000) = 384
Triclinic, *P*1	*D*_x_ = 1.574 Mg m^−^^3^
Hall symbol: -P 1	Melting point = 425–426 K
*a* = 6.3690 (1) Å	Mo *K*α radiation, λ = 0.71073 Å
*b* = 9.2553 (1) Å	Cell parameters from 4607 reflections
*c* = 14.1884 (2) Å	θ = 2.3–30.0°
α = 104.397 (1)°	µ = 2.60 mm^−^^1^
β = 93.748 (1)°	*T* = 100 K
γ = 98.799 (1)°	Block, colorless
*V* = 795.88 (2) Å^3^	0.27 × 0.20 × 0.15 mm

### Data collection

**Table:**

Bruker APEXII CCD area-detector diffractometer	4607 independent reflections
Radiation source: sealed tube	4118 reflections with *I* > 2σ(*I*)
graphite	*R*_int_ = 0.023
φ and ω scans	θ_max_ = 30.0°, θ_min_ = 2.3°
Absorption correction: multi-scan (*SADABS*; Bruker, 2005)	*h* = −8→8
*T*_min_ = 0.541, *T*_max_ = 0.701	*k* = −13→13
16214 measured reflections	*l* = −19→19

### Refinement

**Table:**

Refinement on *F*^2^	Primary atom site location: structure-invariant direct methods
Least-squares matrix: full	Secondary atom site location: difference Fourier map
*R*[*F*^2^ > 2σ(*F*^2^)] = 0.024	Hydrogen site location: inferred from neighbouring sites
*wR*(*F*^2^) = 0.060	All H-atom parameters refined
*S* = 1.02	*w* = 1/[σ^2^(*F*_o_^2^) + (0.029*P*)^2^ + 0.2809*P*] where *P* = (*F*_o_^2^ + 2*F*_c_^2^)/3
4607 reflections	(Δ/σ)_max_ = 0.001
276 parameters	Δρ_max_ = 0.42 e Å^−^^3^
0 restraints	Δρ_min_ = −0.23 e Å^−^^3^

### Special details

**Table:**

Experimental. The crystal was placed in the cold stream of an Oxford Cryosystems Cobra open-flow nitrogen cryostat (Cosier & Glazer, 1986) operating at 100.0 (1) K.
Geometry. All e.s.d.'s (except the e.s.d. in the dihedral angle between two l.s. planes) are estimated using the full covariance matrix. The cell e.s.d.'s are taken into account individually in the estimation of e.s.d.'s in distances, angles and torsion angles; correlations between e.s.d.'s in cell parameters are only used when they are defined by crystal symmetry. An approximate (isotropic) treatment of cell e.s.d.'s is used for estimating e.s.d.'s involving l.s. planes.
Refinement. Refinement of *F*^2^ against ALL reflections. The weighted *R*-factor *wR* and goodness of fit *S* are based on *F*^2^, conventional *R*-factors *R* are based on *F*, with *F* set to zero for negative *F*^2^. The threshold expression of *F*^2^ > σ(*F*^2^) is used only for calculating *R*-factors(gt) *etc*. and is not relevant to the choice of reflections for refinement. *R*-factors based on *F*^2^ are statistically about twice as large as those based on *F*, and *R*- factors based on ALL data will be even larger.

### Fractional atomic coordinates and isotropic or equivalent isotropic displacement parameters (Å^2^)

**Table:**

	*x*	*y*	*z*	*U*_iso_*/*U*_eq_	
Br1	1.13952 (2)	−0.293272 (16)	0.461372 (11)	0.02616 (5)	
O1	0.44064 (16)	−0.15866 (11)	0.80334 (7)	0.0216 (2)	
O2	−0.08941 (16)	0.10051 (10)	0.90599 (7)	0.01977 (19)	
O3	−0.16385 (15)	0.59990 (10)	0.87549 (7)	0.01789 (18)	
O4	0.35439 (15)	0.34158 (10)	0.71793 (7)	0.01744 (18)	
C1	0.6621 (2)	−0.08380 (15)	0.59115 (10)	0.0193 (2)	
C2	0.8032 (2)	−0.13463 (16)	0.52476 (10)	0.0213 (3)	
C3	0.9448 (2)	−0.22219 (14)	0.55005 (10)	0.0184 (2)	
C4	0.9487 (2)	−0.25950 (15)	0.63902 (10)	0.0197 (3)	
C5	0.8037 (2)	−0.21054 (14)	0.70324 (10)	0.0183 (2)	
C6	0.6585 (2)	−0.12259 (13)	0.67994 (9)	0.0153 (2)	
C7	0.4967 (2)	−0.07889 (14)	0.74854 (9)	0.0158 (2)	
C8	0.4106 (2)	0.06017 (14)	0.74754 (9)	0.0168 (2)	
C9	0.2408 (2)	0.08983 (14)	0.79633 (9)	0.0154 (2)	
C10	0.13257 (19)	0.21967 (13)	0.81164 (9)	0.0141 (2)	
C11	−0.0378 (2)	0.22448 (13)	0.87113 (9)	0.0147 (2)	
C12	−0.1436 (2)	0.34794 (14)	0.89404 (9)	0.0155 (2)	
C13	−0.0755 (2)	0.47186 (14)	0.85774 (9)	0.0145 (2)	
C14	0.0926 (2)	0.47405 (14)	0.79966 (9)	0.0153 (2)	
C15	0.19197 (19)	0.34849 (13)	0.77554 (9)	0.0139 (2)	
C16	−0.2346 (2)	0.10739 (16)	0.97956 (10)	0.0194 (3)	
C17	−0.3435 (2)	0.60468 (16)	0.93123 (10)	0.0196 (3)	
C18	0.4171 (2)	0.46747 (15)	0.67761 (11)	0.0198 (3)	
H1	0.562 (3)	−0.026 (2)	0.5736 (13)	0.027 (4)*	
H2	0.802 (3)	−0.108 (2)	0.4646 (14)	0.029 (5)*	
H4	1.047 (3)	−0.321 (2)	0.6562 (13)	0.027 (4)*	
H5	0.809 (3)	−0.2327 (19)	0.7633 (13)	0.024 (4)*	
H8	0.481 (3)	0.1247 (19)	0.7154 (12)	0.017 (4)*	
H9	0.185 (3)	0.0164 (19)	0.8273 (12)	0.021 (4)*	
H12	−0.257 (3)	0.3470 (19)	0.9351 (13)	0.022 (4)*	
H14	0.134 (3)	0.5637 (19)	0.7793 (12)	0.020 (4)*	
H16A	−0.182 (3)	0.1914 (19)	1.0344 (13)	0.022 (4)*	
H16B	−0.233 (3)	0.011 (2)	0.9971 (14)	0.030 (5)*	
H16C	−0.379 (3)	0.1129 (18)	0.9542 (12)	0.016 (4)*	
H17A	−0.383 (3)	0.700 (2)	0.9308 (14)	0.030 (5)*	
H17B	−0.455 (3)	0.5210 (19)	0.8990 (12)	0.020 (4)*	
H17C	−0.302 (3)	0.6012 (18)	0.9976 (13)	0.020 (4)*	
H18A	0.456 (3)	0.561 (2)	0.7289 (14)	0.028 (5)*	
H18B	0.535 (3)	0.444 (2)	0.6459 (14)	0.029 (5)*	
H18C	0.302 (3)	0.4758 (19)	0.6301 (13)	0.023 (4)*	

### Atomic displacement parameters (Å^2^)

**Table:**

	*U*^11^	*U*^22^	*U*^33^	*U*^12^	*U*^13^	*U*^23^
Br1	0.02155 (8)	0.02786 (8)	0.02793 (8)	0.00852 (5)	0.00972 (5)	0.00087 (5)
O1	0.0250 (5)	0.0186 (4)	0.0258 (5)	0.0072 (4)	0.0092 (4)	0.0105 (4)
O2	0.0227 (5)	0.0166 (4)	0.0246 (5)	0.0054 (4)	0.0119 (4)	0.0106 (4)
O3	0.0185 (5)	0.0176 (4)	0.0220 (5)	0.0089 (3)	0.0091 (4)	0.0081 (4)
O4	0.0197 (5)	0.0152 (4)	0.0211 (4)	0.0062 (3)	0.0102 (4)	0.0078 (3)
C1	0.0199 (6)	0.0216 (6)	0.0183 (6)	0.0097 (5)	0.0017 (5)	0.0053 (5)
C2	0.0223 (7)	0.0260 (7)	0.0170 (6)	0.0081 (5)	0.0033 (5)	0.0054 (5)
C3	0.0158 (6)	0.0168 (6)	0.0204 (6)	0.0033 (5)	0.0036 (5)	0.0002 (5)
C4	0.0178 (6)	0.0161 (6)	0.0271 (7)	0.0065 (5)	0.0030 (5)	0.0067 (5)
C5	0.0196 (6)	0.0161 (6)	0.0213 (6)	0.0046 (5)	0.0031 (5)	0.0076 (5)
C6	0.0154 (6)	0.0118 (5)	0.0180 (6)	0.0021 (4)	0.0021 (4)	0.0027 (4)
C7	0.0168 (6)	0.0140 (5)	0.0164 (6)	0.0031 (4)	0.0018 (4)	0.0030 (4)
C8	0.0203 (6)	0.0137 (5)	0.0176 (6)	0.0044 (5)	0.0042 (5)	0.0051 (4)
C9	0.0171 (6)	0.0131 (5)	0.0158 (6)	0.0022 (4)	0.0013 (4)	0.0039 (4)
C10	0.0143 (6)	0.0141 (5)	0.0137 (5)	0.0023 (4)	0.0021 (4)	0.0031 (4)
C11	0.0155 (6)	0.0137 (5)	0.0149 (5)	0.0012 (4)	0.0019 (4)	0.0049 (4)
C12	0.0143 (6)	0.0175 (6)	0.0155 (6)	0.0037 (4)	0.0038 (4)	0.0047 (4)
C13	0.0143 (6)	0.0149 (5)	0.0149 (5)	0.0047 (4)	0.0011 (4)	0.0036 (4)
C14	0.0171 (6)	0.0147 (5)	0.0157 (6)	0.0040 (4)	0.0035 (4)	0.0057 (4)
C15	0.0135 (5)	0.0151 (5)	0.0132 (5)	0.0027 (4)	0.0030 (4)	0.0036 (4)
C16	0.0197 (6)	0.0206 (6)	0.0203 (6)	0.0026 (5)	0.0082 (5)	0.0087 (5)
C17	0.0172 (6)	0.0228 (6)	0.0212 (6)	0.0076 (5)	0.0070 (5)	0.0061 (5)
C18	0.0229 (7)	0.0165 (6)	0.0244 (7)	0.0062 (5)	0.0125 (5)	0.0093 (5)

### Geometric parameters (Å, °)

**Table:**

Br1—C3	1.8963 (13)	C8—H8	0.920 (17)
O1—C7	1.2321 (15)	C9—C10	1.4524 (17)
O2—C11	1.3627 (14)	C9—H9	0.936 (17)
O2—C16	1.4351 (15)	C10—C11	1.4171 (17)
O3—C13	1.3634 (15)	C10—C15	1.4205 (16)
O3—C17	1.4326 (16)	C11—C12	1.3943 (17)
O4—C15	1.3591 (14)	C12—C13	1.3927 (17)
O4—C18	1.4364 (15)	C12—H12	0.957 (18)
C1—C2	1.3910 (19)	C13—C14	1.3937 (17)
C1—C6	1.3939 (18)	C14—C15	1.3870 (17)
C1—H1	0.953 (18)	C14—H14	0.950 (17)
C2—C3	1.388 (2)	C16—H16A	0.953 (18)
C2—H2	0.943 (19)	C16—H16B	0.985 (19)
C3—C4	1.3888 (19)	C16—H16C	0.980 (17)
C4—C5	1.3858 (19)	C17—H17A	0.956 (18)
C4—H4	0.965 (18)	C17—H17B	0.961 (17)
C5—C6	1.3969 (18)	C17—H17C	0.971 (17)
C5—H5	0.924 (18)	C18—H18A	0.971 (19)
C6—C7	1.4954 (17)	C18—H18B	0.93 (2)
C7—C8	1.4768 (17)	C18—H18C	0.987 (18)
C8—C9	1.3486 (18)		
			
C11—O2—C16	118.40 (10)	O2—C11—C12	122.08 (11)
C13—O3—C17	118.06 (10)	O2—C11—C10	115.23 (11)
C15—O4—C18	117.90 (10)	C12—C11—C10	122.69 (11)
C2—C1—C6	121.15 (12)	C13—C12—C11	118.12 (11)
C2—C1—H1	118.8 (11)	C13—C12—H12	121.9 (10)
C6—C1—H1	120.0 (11)	C11—C12—H12	120.0 (10)
C3—C2—C1	118.35 (13)	O3—C13—C12	123.91 (11)
C3—C2—H2	121.6 (11)	O3—C13—C14	114.39 (11)
C1—C2—H2	120.1 (11)	C12—C13—C14	121.70 (11)
C2—C3—C4	121.82 (13)	C15—C14—C13	119.29 (11)
C2—C3—Br1	119.49 (10)	C15—C14—H14	123.6 (10)
C4—C3—Br1	118.70 (10)	C13—C14—H14	117.1 (10)
C5—C4—C3	118.94 (12)	O4—C15—C14	122.54 (11)
C5—C4—H4	119.5 (11)	O4—C15—C10	115.72 (10)
C3—C4—H4	121.5 (11)	C14—C15—C10	121.72 (11)
C4—C5—C6	120.70 (12)	O2—C16—H16A	110.1 (11)
C4—C5—H5	118.9 (11)	O2—C16—H16B	102.7 (11)
C6—C5—H5	120.4 (11)	H16A—C16—H16B	110.8 (15)
C1—C6—C5	119.01 (12)	O2—C16—H16C	112.1 (10)
C1—C6—C7	121.73 (11)	H16A—C16—H16C	110.4 (14)
C5—C6—C7	119.20 (11)	H16B—C16—H16C	110.5 (14)
O1—C7—C8	122.63 (12)	O3—C17—H17A	103.5 (11)
O1—C7—C6	119.49 (11)	O3—C17—H17B	108.7 (10)
C8—C7—C6	117.87 (11)	H17A—C17—H17B	112.0 (15)
C9—C8—C7	119.33 (11)	O3—C17—H17C	110.5 (10)
C9—C8—H8	123.4 (10)	H17A—C17—H17C	110.8 (15)
C7—C8—H8	117.2 (10)	H17B—C17—H17C	111.1 (14)
C8—C9—C10	130.75 (12)	O4—C18—H18A	111.0 (11)
C8—C9—H9	115.1 (11)	O4—C18—H18B	104.2 (11)
C10—C9—H9	114.1 (11)	H18A—C18—H18B	110.1 (15)
C11—C10—C15	116.44 (11)	O4—C18—H18C	110.9 (10)
C11—C10—C9	118.69 (11)	H18A—C18—H18C	110.4 (15)
C15—C10—C9	124.78 (11)	H18B—C18—H18C	110.0 (15)
			
C6—C1—C2—C3	−1.6 (2)	C15—C10—C11—O2	−179.05 (11)
C1—C2—C3—C4	0.0 (2)	C9—C10—C11—O2	−2.38 (17)
C1—C2—C3—Br1	−179.94 (10)	C15—C10—C11—C12	−0.09 (18)
C2—C3—C4—C5	1.4 (2)	C9—C10—C11—C12	176.58 (12)
Br1—C3—C4—C5	−178.66 (10)	O2—C11—C12—C13	177.93 (11)
C3—C4—C5—C6	−1.2 (2)	C10—C11—C12—C13	−0.96 (19)
C2—C1—C6—C5	1.8 (2)	C17—O3—C13—C12	−2.84 (18)
C2—C1—C6—C7	−175.19 (12)	C17—O3—C13—C14	177.45 (11)
C4—C5—C6—C1	−0.4 (2)	C11—C12—C13—O3	−179.38 (11)
C4—C5—C6—C7	176.71 (12)	C11—C12—C13—C14	0.31 (19)
C1—C6—C7—O1	151.49 (13)	O3—C13—C14—C15	−178.89 (11)
C5—C6—C7—O1	−25.51 (18)	C12—C13—C14—C15	1.40 (19)
C1—C6—C7—C8	−28.65 (18)	C18—O4—C15—C14	−2.80 (18)
C5—C6—C7—C8	154.35 (12)	C18—O4—C15—C10	178.51 (11)
O1—C7—C8—C9	−10.8 (2)	C13—C14—C15—O4	178.88 (11)
C6—C7—C8—C9	169.35 (12)	C13—C14—C15—C10	−2.52 (19)
C7—C8—C9—C10	176.36 (12)	C11—C10—C15—O4	−179.45 (10)
C8—C9—C10—C11	−176.74 (13)	C9—C10—C15—O4	4.10 (18)
C8—C9—C10—C15	−0.4 (2)	C11—C10—C15—C14	1.86 (18)
C16—O2—C11—C12	−9.31 (18)	C9—C10—C15—C14	−174.59 (12)
C16—O2—C11—C10	169.65 (11)		

### Hydrogen-bond geometry (Å, °)

**Table:**

*D*—H···*A*	*D*—H	H···*A*	*D*···*A*	*D*—H···*A*
C5—H5···O3^i^	0.925 (18)	2.497 (18)	3.3572 (17)	154.9 (15)
C8—H8···O4	0.920 (18)	2.268 (18)	2.8103 (16)	117.2 (14)
C9—H9···O1	0.936 (18)	2.449 (19)	2.8128 (17)	103.1 (13)
C9—H9···O2	0.936 (18)	2.269 (19)	2.6960 (16)	107.1 (14)
C18—H18A···O1^ii^	0.968 (19)	2.566 (19)	3.4518 (18)	152.1 (15)
C17—H17C···Cg1^iii^	0.971 (17)	2.754 (18)	3.6601 (14)	155.6 (14)

Symmetry codes: (i) *x*+1, *y*−1, *z*;  (ii) *x*, *y*+1, *z*; (iii) −*x*, −*y*+1, −*z*+2.
